# Hypoxic preconditioning‐induced autophagy enhances survival of engrafted endothelial progenitor cells in ischaemic limb

**DOI:** 10.1111/jcmm.13167

**Published:** 2017-04-04

**Authors:** Pei Zhou, Yu‐zhen Tan, Hai‐jie Wang, Guo‐dong Wang

**Affiliations:** ^1^ Department of Anatomy, Histology and Embryology Shanghai Medical School of Fudan University Shanghai China

**Keywords:** endothelial progenitor cells, hypoxia, autophagy, fibrin, survival, transplantation

## Abstract

Recent clinical studies have suggested that endothelial progenitor cells (EPCs) transplantation provides a modest benefit for treatment of the ischaemic diseases such as limb ischaemia. However, cell‐based therapies have been limited by poor survival of the engrafted cells. This investigation was designed to establish optimal hypoxia preconditioning and evaluate effects of hypoxic preconditioning‐induced autophagy on survival of the engrafted EPCs. Autophagy of CD34^+^ VEGFR‐2^+^ EPCs isolated from rat bone marrow increased after treatment with 1% O_2_. The number of the apoptotic cells in the hypoxic cells increased significantly after autophagy was inhibited with 3‐methyladenine. According to balance of autophagy and apoptosis, treatment with 1% O_2_ for 2 hrs was determined as optimal preconditioning for EPC transplantation. To examine survival of the hypoxic cells, the cells were implanted into the ischaemic pouch of the abdominal wall in rats. The number of the survived cells was greater in the hypoxic group. After the cells loaded with fibrin were transplanted with intramuscular injection, blood perfusion, arteriogenesis and angiogenesis in the ischaemic hindlimb were analysed with laser Doppler‐based perfusion measurement, angiogram and the density of the microvessels in histological sections, respectively. Repair of the ischaemic tissue was improved significantly in the hypoxic preconditioning group. Loading the cells with fibrin has cytoprotective effect on survival of the engrafted cells. These results suggest that activation of autophagy with hypoxic preconditioning is an optimizing strategy for EPC therapy of limb ischaemia.

## Introduction

Critical limb ischaemia (CLI) is a clinical consequence of peripheral arterial disease (PAD, such as diabetes), which is characterized by chronic ischaemic rest pain, ulcer or gangrene in one or both lower limbs. The incidence of CLI has been estimated to be 500–1000 per million/year in the European and North American population. An estimated 5–10% of PAD patients aged >50 years develop CLI ≤5 years. Patients with CLI are estimated to face >30% 1‐year amputation rates and >20% 1‐year mortality 1. The pathophysiology of CLI is a chronic and complex process that affects the macrovascular and microvascular systems, as well as surrounding tissues 2. Chronic ischaemia from macroscopic disease leads to alterations in structure and function of endothelial cells and alterations in pressure unloading. These endothelial changes result in increased free radical production, platelet activation and leucocyte adhesion, all of which lead to microthrombi formation and microvascular obstruction 3.

Traditional CLI treatments include medical therapy, endovascular intervention, open surgery or amputation. In recent years, the therapies promoting revascularization such as angiogenesis and arteriogenesis have been intensely studied 4. Compared with local administration of growth factors such as vascular endothelial growth factor (VEGF), cell‐based therapy is more safe and effective 5. Endothelial progenitor cells (EPCs) are a population of cells that are located in bone marrow, peripheral blood and cord blood and can proliferate and differentiate into mature endothelial cells. In pathologic conditions, EPCs are mobilized into the local tissue to participate in formation of new vessels and establishment of collateral anastomosis 6, 7, 8. However, the number and function of EPCs in some patients with cardiovascular diseases are reduced as a consequence of exposure to risk factors 9. The most common risk factors for the development of advanced PAD and CLI are diabetes, smoking, old age and chronic kidney disease 3. Therefore, it is necessary to transplant autologous or allogenic EPCs to promote angiogenesis of the ischaemic tissue, compensating for a shortage of endogenous EPCs. Recently, clinical trials show that intramuscular EPC transplantation provides a novel therapy in patients with CLI. After transplantation of EPCs in patients with CLI, reperfusion of the lower limb increases, rest and walking pain decreases and amputation rate is reduced 10, 11, 12. The implanted cells salvage the ischaemic limb by contributing to vasculogenesis, angiogenesis and arteriogenesis 13. Unfortunately, cell‐based therapies have been limited by poor survival and differentiation of the engrafted cells within ischaemic and hypoxic microenvironment 14. Therefore, optimizing EPC transplantation with preconditioning strategy is beneficial to tolerance of the implanted cells in the local hostile environment. In recent years, effectiveness of hypoxia preconditioning on cell therapy of the ischaemic limb has been explored intensively. Transplantation of hypoxia‐preconditioned mesenchymal stem cells (MSCs) augments revascularization and repair of the ischaemic muscle 15, 16. Hypoxia‐treated EPCs increase neovascularization efficacy after transplantation into the ischaemic limb 17. Hypoxia inhibits EPC senescence; hypoxia‐preconditioned old human EPCs restore therapeutic potential in vasculogenesis of the ischaemic hindlimb 18. However, desirable hypoxic preconditioning for EPC transplantation has not been defined. Therefore, it is useful to select optimal hypoxia conditions and elucidate mechanisms of hypoxic preconditioning for enhancing the survival and differentiation of the implanted cells.

Autophagy plays critical roles in self‐renewal, differentiation and ageing of stem cells 19, 20, 21. In addition, autophagy is implicated in proliferation and differentiation of EPCs 22. Autophagy is a cellular pathway involved in protein and organelle degradation to get rid of the degenerated or senescent organelles and aggregation‐prone proteins and supply energy for cellular activities. Autophagy may be divided into macroautophagy, microautophagy and chaperone‐mediated autophagy based on the pathways by which cargos are delivered into lysosomes 23. Autophagy occurs at basal, constitutive levels and is activated under pathological conditions 24. Autophagy‐mediated cytoprotective effects include the ability of autophagy to buffer against starvation, protect against apoptotic insults and clear mitochondria and aggregate‐prone proteins 25. We suggest that hypoxia activates autophagy, promoting EPC survival by inhibiting apoptosis 22. It will be important to fine‐tune the balance between the induction of autophagy to promote EPC survival under low O_2_ tension and the excessive hypoxia that leads to cellular stress and subsequent apoptosis for enhancing EPC survival following transplantation 26.

This investigation was designed to select an optimal time‐point of hypoxic preconditioning and investigate effects of hypoxia‐induced macroautophagy (hereafter termed ‘autophagy’) on survival of the engrafted EPCs. According to balance of autophagy and apoptosis induced with hypoxia, treatment with 1% O_2_ for 2 hrs was determined as optimal preconditioning of EPCs. The survival of the hypoxic cells was examined with implantation of the cells into the ischaemic pouches of the abdominal wall in rats. Efficiency of the engrafted hypoxic cells in promoting neovascularization and tissue repair was evaluated in rat ischaemic hindlimb models. Moreover, reliability of fibrin in delivery of EPCs was investigated. Here, we report that hypoxia‐preconditioned cells enhance arteriogenesis and angiogenesis and restore blood perfusion of the ischaemic hindlimb significantly after transplantation.

## Materials and methods

### Isolation and identification of CD34^+^VEGFR‐2^+^ EPCs

Sprague Dawley (SD) rats were anaesthetized with intraperitoneal injection of ketamine (80 mg/kg) and xylazine (3 mg/kg) and killed with pulling the neck. The protocol was approved by the Institutional Animal Care Committee of Fudan University. The mononuclear cells in the bone marrow of 100 rats (30–50 g) were isolated with percoll solution (GE Healthcare, Leics, UK) using gradient centrifugation. CD34^+^VEGFR‐2^+^ cells in the mononuclear cells were sorted by a flow cytometry (Beckman Coulter, Fullerton, CA, USA). The cells were seeded into gelatin‐coated culture dishes at a density of 1 × 10^5^ cells/ml and incubated in DMEM supplemented with 10 ng/ml VEGF (Peprotech, Rocky Hill, NJ, USA) and 15% FBS. Differentiation of the cells into the endothelial cells was determined by acquisition of CD31 expression using immunostaining after induction for 2 weeks. The EPCs incubated for 7 days were used for the following experiments.

### Analysis of survival and viability of the cells

CD34^+^VEGFR‐2^+^ EPCs were divided into control and hypoxia groups and were incubated for 30 min., 1 hr, 2 hrs, 4 hrs and 6 hrs, respectively. In the hypoxia groups, the culture dishes were put into a hypoxia chamber (Billups‐Rothenberg, San Diego, CA, USA) flushed with a gas mixture of 1% O_2_, 5% CO_2_ and 94% N_2_. Concentration of oxygen was measured with an oxygen analyser (Billups‐Rothenberg) 27. After hypoxia treatment, the cells were collected and incubated with 1 μg/ml FITC‐Annexin V and 1 μg/ml propidium iodide (PI; BD Biosciences, San Jose, CA, USA) for 20 min. The apoptotic cells in the cells were analysed by flow cytometry.

In following experiments, the cells were divided into control, hypoxia and 3‐MA groups. In hypoxia group, the cells were treated with hypoxia (1% O_2_) for 2 hrs. In 3‐MA group, the cells were pre‐treated with 5 mM 3‐MA (3‐methyladenine; Sigma‐Aldrich, St. Louis, MO, USA) for 1 hr and then treated with hypoxia for 2 hrs. 3‐MA, an inhibitor of class III PI3K, inhibits formation of autophagosome 28. Apoptosis of the cells was examined with ethidium bromide and acridine orange (EB/AO) staining 29. The percentages of the apoptotic cells and necrotic cells were counted in five fields randomly. Cell viability was detected with Cell Counting Kit‐8 (CCK‐8; Dojindo, Kumamoto, Japan). After treatment with 100 μl CCK‐8 (1:10 in DMEM), the cells continued to be incubated for 1 hr. The supernatant was transferred into 96‐well plate. Cell viability was reflected by OD_450_ with a microplate reader (Tecan Infinite 200, Mannedort, Switzerland).

To evaluate cytoprotective effect of fibrin gel, apoptosis of the hypoxic cells was examined with EB/AO staining. The cells were suspended with 10 mg/ml fibrinogen (Sigma‐Aldrich), then the same amount of 10 IU/ml thrombin (Sigma‐Aldrich) was mixed with gentle shake immediately. The cells in fibrin gel were treated with 1% O_2_ for 4 hrs.

### RT‐qPCR analysis

Total RNA of the cells was extracted using the TRIzol Reagent Kit (Invitrogen, Carlsbad, CA, USA). The quality of total RNA was verified with ultraviolet absorbance spectrophotometry at 260 and 280 nm. Reversed transcription was reacted using Primescript RT Reagent Kit with gDNA Eraser (TAKARA Biotechnology, Otsu, Japan) based on the manufacturer's instructions as follows: 37°C for 15 min., 85°C for 5 sec. and 4°C forever before taking out. cDNA was stored at −30°C. The level of VEGFR‐2 mRNA expression was quantified by RT‐qPCR. The sequences of VEGFR‐2 primers were as follows: (F)5′‐TTTGTGGTTTATAAGTCTCAGGACG‐3′, (R)5′‐TCAATGTGTAAGCCAGGGTAAGGGG‐3′.

### Enzyme‐linked immunosorbent assay (ELISA)

The cells were divided into control, hypoxia and 3‐MA groups. In hypoxia and 3‐MA groups, the cells were treated with 1% O_2_ for 2, 4, 8 and 12 hrs. The levels of VEGF in the supernatants of each group at different time‐points were detected using VEGF ELISA Kit (Boster, Wuhan, China) according to the manufacturer's protocol. Absorbance values were measured at 450 nm with a microplate reader.

### Western blotting

Cells were divided into control and hypoxia groups. In hypoxia groups were incubated in 1% O_2_ for 30 min., 1 hr, 2 hrs, 4 hrs and 6 hrs. After total protein in each group was extracted with cell lysate (volume ratio of RAPI to PMSF was 100:1) and denatured at 95°C for 5 min., proteins were separated with 15% SDS–polyacrylamide gel and blotted onto a PVDF membrane. Non‐specific binding sites were blocked using 5% skim milk. Blots were probed with polyclonal rabbit anti‐rat LC3 (microtubule‐associated protein 1 light chain 3) antibody (1:500; Novus, Littleton, CO, USA) at 4°C overnight followed by anti‐rabbit IgG HRP‐linked antibody (1:4000; Cell Signaling, Danvers, MA, USA) and mouse anti‐rat β‐actin antibody (1:4000; Sigma‐Aldrich) followed by anti‐mouse IgG HRP‐linked antibody (1:4000; Cell Signaling), respectively. Then, the proteins were visualized by Clarity Western ECL Blotting Substrate (BIO‐RAD, Hercules, CA, USA)and scanned with ChemiDocTM MP system. The ratio of LC3‐II/LC3‐I was analysed using ImageJ (National Institutes of Health, Bethesda, MD, USA).

### LC3 immunostaining

LC3 expresses mainly on autophagosome and is used as a specific marker for autophagic structures 30. The cells were treated with hypoxia for 2 hrs and then were fixed with 4% paraformaldehyde. After being blocked with goat serum, the cells were incubated with polyclonal antibody against LC3 (1:200; Novus) at 4°C overnight and afterwards with Alexa Fluor 488 goat anti‐rabbit IgG (1:200; Jackson) at 37°C for 30 min. The nuclei were counterstained with DAPI. LC3‐positive puncta were viewed with a confocal laser scanning microscope and counted in five fields randomly for three coverslips in each group.

### Transmission and scanning electron microscopies

Cells treated with hypoxia for 2 hrs were prefixed with 2.5% glutaraldehyde at 4°C overnight and post‐fixed with 1% buffered osmium tetroxide. Then, the specimens went dehydrated with graded ethanol and two final 15min. rinses in 100% ethanol. After being embedded, the specimens were solidified in 37, 45 and 60°C. Ultrathin sections were prepared and stained with uranyl acetate and lead citrate. The autophagic structures in the cells were examined with a transmission electron microscope (CM120; Philips, Eindhoven, Holland). Sectional areas of the autophagic structures were measured with Photoshop software, and the ratios of the cross‐sectional areas of the autophagic structures to that of the cytoplasm were calculated. The autophagic structures were examined in 200 cells for each group. In another experiment, the cells in fibrin glue were incubated for 1 hr. Distribution of the cells in fibrin glue was examined with transmission electron microscopy.

For scanning electron microscopy, the cells were seeded on coverslips coated with fibrin and incubated for 1 hr. The specimens were fixed with 2.5% glutaraldehyde, dried under vacuum and coated with gold‐palladium. Morphologic features of the cells on fibrin glue were investigated using a scanning electron microscope (Hitachi SU8010, Tokyo, Japan).

### Implantation of the cells into abdominal pouches

To access the survival of hypoxia‐preconditioned cells in the ischaemic tissue, the cells were implanted into abdominal pouches. The cells were treated with 5 μM DiI for 20 min., then suspended with 20 ng/ml fibrinogen and 50 IU/ml thrombin, respectively, and seeded on the poriferous polyethylene terephthalate membrane (pore size, 8 μm) removed from the transwell (Becton Dickinson, Franklin Lakes, NJ, USA). Nine male rats were divided in control, hypoxia 2hrs and 3‐MA groups (three rats for each group). 2‐cm‐long incision on the skin of the median line of the anterior abdominal wall was made, and then the superficial fascia at both sides was bluntly dissected with a forceps to create two ischaemic pouches. The vessels at the subcutaneous pouches were ligated. After incubation for 30 min., the cell‐loaded membranes were implanted into the pouches. At 12 or 24 hrs after implantation, the membranes were harvested, and the survived cells (DiI‐labelled cells) were examined with a fluorescent microscope.

### Transplantation of the cells into the ischaemic hindlimbs

The model of hindlimb ischaemia was established by ligation of the femoral artery according to the previous method 31. In brief, the left femoral artery of male rats (8 weeks old) was ligated below the inguinal ligament and dissected free along its entire length. Major branches of the artery were also ligated. The right hindlimb was served as a control in each rat. Thirty rats were divided into normal, control, PBS, EPC, hypoxia, fibrin and 3‐MA groups. The right hindlimb was regarded as normal. In control group, only the models of hindlimb ischaemia were examined. In PBS group, 100 μl PBS was injected into muscles of the thigh at five different points. In EPC group, 5 × 10^5^ cells in 100 μl PBS were transplanted by intramuscular injection. In hypoxia group, the cells were pretreated with hypoxia for 2 hrs. In fibrin group, 50 μl fibrinogen (10 mg/ml) containing hypoxia‐preconditioned EPCs and 50 μl thrombin (10 IU/ml) were simultaneously injected using a Duploject syringe. In 3‐MA group, hypoxia‐treated EPCs were inhibited with 3‐MA, and the cells were transplanted with same method as fibrin group.

### Laser Doppler imaging

Laser Doppler imaging is a reliable tool to assess relative limb perfusion if the region of interest is precisely confined to the plantar sole 32. The limb blood flow of the rats was evaluated with a laser Doppler perfusion image analyser at day 0, 3, 7, 14 and 21, respectively, after cell transplantation. Low‐to‐no flow was displayed as dark blue, whereas high blood flow was displayed as red. Before surgery, blood flow of each limb was measured to ensure blood supply of legs was normal, and immediate post‐surgery laser Doppler imaging scans at day 0 could confirm a successful ligation of the femoral artery. Blood flow of the hindlimbs in each rat was scanned twice, and the value of blood flow was expressed as the average of the percentages of ischaemic (left)/normal (right) limb flow.

### Angiography

Formation of collateral vessels in ischaemic hindlimbs was analysed by angiography with Quantum FX microCT (PerkinElmer, Waltham, MA, USA) at day 21 after cell transplantation. The abdominal wall of rats was incised at the midline. A I.V. catheter connecting with a tube was inserted into abdominal aorta. After injection of non‐ionic contrast medium (Ultravist 370; BAYER, Whippany, NJ, USA), the ischaemic area of the limb was scanned immediately, and 3D images of the vessels were reconstructed with *Z*‐axis cross‐sectional slices. The vessels were identified after thresholding, and vascular volume was evaluated using Caliper Analyze 12.0 (Analyze Direct, Overland Park, KS, USA).

### Immunohistochemistry

To determine angiogenesis in the ischaemic hindlimb and repair of the ischaemic skeletal muscles, semimembranosus and gastrocnemius were removed at day 21 after cell transplantation and embedded in optimal cutting temperature compound. The microvessels in frozen sections were examined using CD31 immunostaining. In counting capillaries, three fields were selected at each section for three sections. In order to avoid overestimation or underestimation of capillary density, the muscle fibres stained with dystrophin were used to adjust the number of capillaries 33. To determine whether the transplanted EPCs incorporate into the new‐formed vessels, the cells were transfected with Lenti‐GFP virus (Sangon Biotech, Shanghai, China) at a MOI of 30 for 72 hrs before transplantation. At day 14 after transplantation, location of EPC‐derived endothelial cells at microvessels was examined with GFP (rabbit anti‐GFP antibody, 1:100) and CD31 immunostaining.

### Statistical analysis

The statistical analysis was conducted with *t*‐test and one‐way ANNOVA using SPSS 17.0 software (SPSS, Chicago, IL, USA). Quantitative data are presented as means ± standard deviation of at least three experiments. A value of *P* < 0.05 was considered statistically significant between different groups.

## Results

### Differentiation of CD34^+^VEGFR‐2^+^ EPCs

**Figure 1:**
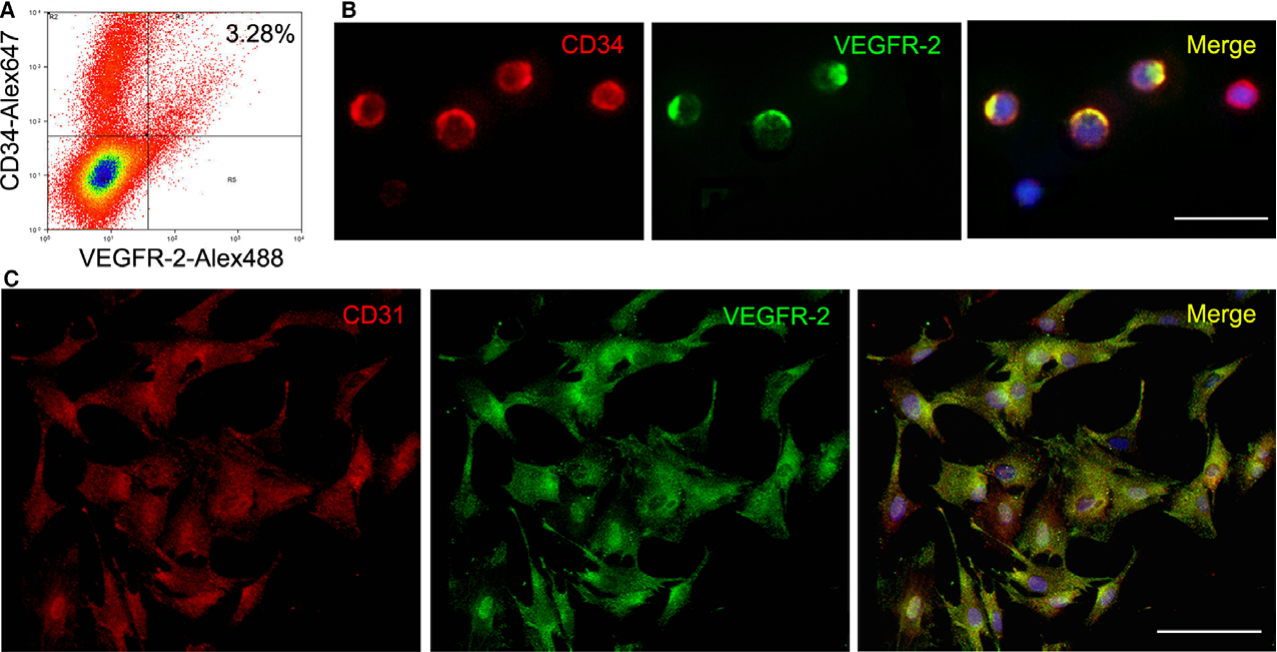
Characteristics of CD34^+^/VEGFR‐2^+^ EPCs. (**A**) EPC phenotype of the mononuclear cells analysed by dual‐colour flow cytometry. CD34^+^ VEGFR‐3^+^ cells present in the mononuclear cells isolated from bone marrow of rats. (**B**) Immunostaining of CD34^+^ VEGFR‐2^+^ cells in the mononuclear cells. Bar = 25 μm. (**C**) Differentiation of CD34^+^ VEGFR‐2^+^ cells towards endothelial cells. At two weeks after induction with VEGF, the cells differentiate into CD31^+^ VEGFR‐2^+^ endothelial cells. Bar = 100 μm. EPC: endothelial progenitor cells; VEGF: vascular endothelial growth factor.

There was a population of cells expressing CD34 and VEGFR‐2 in the mononuclear cells isolated from the bone marrow of the rats. According to flow cytometric analysis, the frequency of CD34^+^VEGFR‐2^+^ cells was 3.28% of the mononuclear cells. At two weeks after induction with VEGF, most of the cells differentiated into CD31^+^VEGFR‐2^+^ endothelial cells (Fig. 1).

### Optimal hypoxia preconditioning

**Figure 2:**
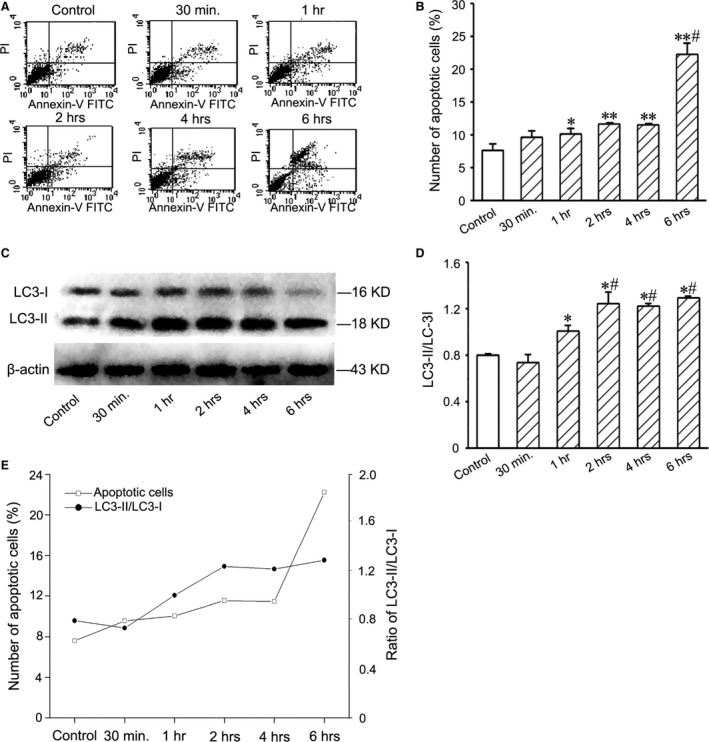
Apoptosis and LC3 expression of EPCs after treatment with 1% O_2_. (**A**) Typical quadrantal diagrams of flow cytometric analysis of the apoptotic cells. (**B**) Statistic result of numbers of the apoptotic cells. After hypoxia treatment, the number of the apoptotic cells increases. *P < 0.05 and **P < 0.01 *versus* control group, #*P* < 0.05 *versus* hypoxia 30 min., 1, 2 and 4hrs groups. (**C**) Western blotting of LC3 in the cells. The levels of LC3‐II expression in the hypoxic cells increased. (**D**) Statistic result of LC3‐II/LC3‐I ratios. At 2 hrs after hypoxia treatment, LC3‐II/LC3‐I ratio reaches the plateau. *P < 0.05 *versus* control group, #*P* < 0.05 *versus* hypoxia 30min. and 1hr groups. (**E**) The curves of the numbers of the apoptotic cells and LC3‐II/LC3‐I ratios after hypoxia treatment. EPC: endothelial progenitor cells.

After treatment with hypoxia for 30 min., 1 hr, 2 hrs, 4 hrs and 6 hrs, the apoptotic cells increased gradually (Fig. 2A and B). LC3‐II expression of the cells was enhanced after hypoxia treatment. Ratio of LC3‐II/LC3‐I reached the plateau at 2 hrs after treatment (Fig. 2C and D). According to time course of apoptosis and autophagy activation of the hypoxia cells, treatment with 1% O_2_ for 2 hrs was selected as an optimal time of hypoxic preconditioning for EPC transplantation (Fig. 2E).

### Changes in autophagic structures after treatment with hypoxia

**Figure 3:**
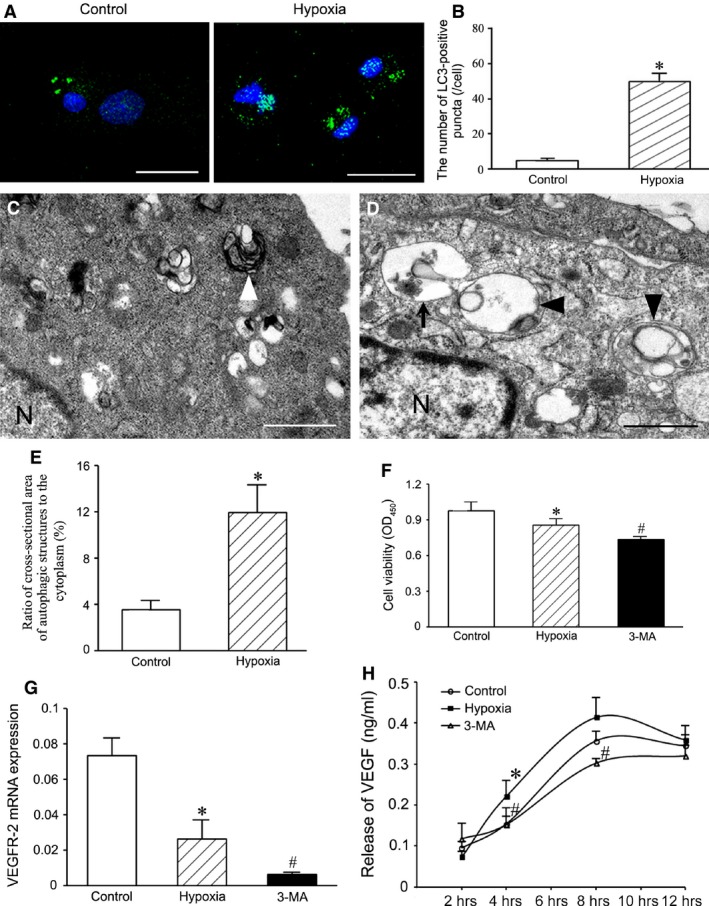
Autophagy, survival, VEGF‐2 mRNA expression and VEGF release of the cells treated with hypoxia for 2 hrs. (**A**) The autophagic structures demonstrated with LC3 immunostaining. Bar = 20 μm. (**B**) Statistic result of LC3‐positive puncta. The puncta in the cells of hypoxia group increase. *P < 0.01 *versus* control group. (**C** and **D**) Representative autophagic ultrastructures. The autophagic ultrastructures in the hypoxic cell (**D**) is more than that in the normal cell (**C**). Arrow shows a cup‐like autophagosome precursor with double membranes; arrowheads indicate autolysosomes containing one or more than one autophagosomes with single membrane. N, nucleus. Bar = 1 μm. (**E**) Statistic result of ratios of the cross‐sectional areas of the autophagic structures to that of the cytoplasm. *P < 0.05 *versus* control group. (**F**) Cell viability detected with CCK‐8. After autophagy is inhibited with 3‐MA, viability of hypoxia‐conditioned cells decreases. *P < 0.01 *versus* control group, #*P* < 0.05 *versus* hypoxia group. (**G**) Expression of VEGFR‐2 mRNA. The level of VEGFR‐2 mRNA expression in the hypoxic cells decreases after treatment with 3‐MA. *P < 0.05 *versus* control group, #*P* < 0.05 *versus* hypoxia group. (**H**) Release of VEGF from the cells. In hypoxia group, VEGF release at 4, 6 and 8 hrs after treatment with hypoxia increases. After autophagy is inhibited with 3‐MA, VEGF release from the hypoxic cells is reduced. *P < 0.05 *versus* control group, #*P* < 0.05 *versus* hypoxia group. VEGF: vascular endothelial growth factor.

Autophagic structures labelled with LC3 immunostaining were round or oval puncta in cytoplasm (Fig. 3A). The number of LC3‐positive puncta in the cells was greater in hypoxia (1% O_2_ for 2 hrs) group than in control group (Fig. 3B). Representative autophagic ultrastructures in hypoxia‐treated cells are shown in Figure 3C and D. After hypoxia treatment, the number of autophagosome precursors, autophagosomes and autolysosomes increased. Ratios of the cross‐sectional areas of the autophagic ultrastructures to that of the cytoplasm in hypoxia group were significantly higher than that in control group (Fig. 3E).

### Cytoprotective effect of hypoxia‐induced autophagy

The apoptotic cells in the hypoxia (1% O_2_ for 2 hrs) group were more than those in the control group. Compared with the hypoxia group, the number of the apoptotic cells in 3‐MA group was greater significantly (Fig. S1). In hypoxia condition, viability of the cells was reduced. After pre‐treatment with 3‐MA, viability of the hypoxic cells decreased significantly (Fig. 3F). VEGFR‐2 mRNA expression of the cells exposed to hypoxia condition decreased. In 3‐MA group, the level of VEGFR‐2 mRNA expression was lower than that in hypoxia group (Fig. 3G). In ELISA, concentration of VEGF in the supernatant of cells at 4, 6 and 8 hrs after treatment with hypoxia was higher than in control group. Compared with hypoxia group, VEGF release in 3‐MA group was reduced significantly (Fig. 3H).

### Effect of fibrin on cell survival in hypoxia condition

**Figure 4:**
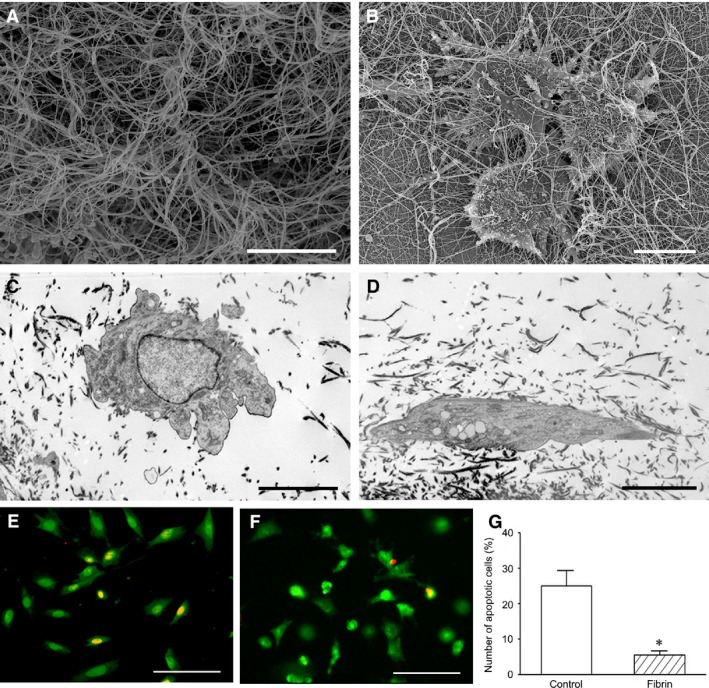
Growth and survival of the cells in fibrin. (**A** and **B**) Scanning electron micrographs of fibrin nanofibres and the cells on them. The cells grow well on the nanofibres. Bar = 5 μm. (**C** and **D**) Transmission electron micrographs of the cells in fibrin. Bar = 5 μm. (**E** and **F**) Protection of the cells by fibrin in hypoxia condition. There are more the survived cells in fibrin group (**F**) than in control group (**E**) after hypoxia treatment. EB/AO staining. Bar = 100 μm. (**G**) Statistic analysis of the number of the apoptotic cells. *P < 0.05 *versus* control group.

In the images of scanning electron microscope, fibrin constituted a delicate fibrous network. The cells spread well on the fibrous network. The pseudopods of the cells extended along the fibres (Fig. 4A and B). In the images of transmission electron microscope, the cells seeded in three‐dimensional fibrin gel grew well (Fig. 4C and D). Compared with control group, the number of the apoptotic cells in fibrin group was less significantly in hypoxia condition (Fig. 4E–G).

### Survival of hypoxia‐preconditioned cells after implantation into abdominal pouches

**Figure 5:**
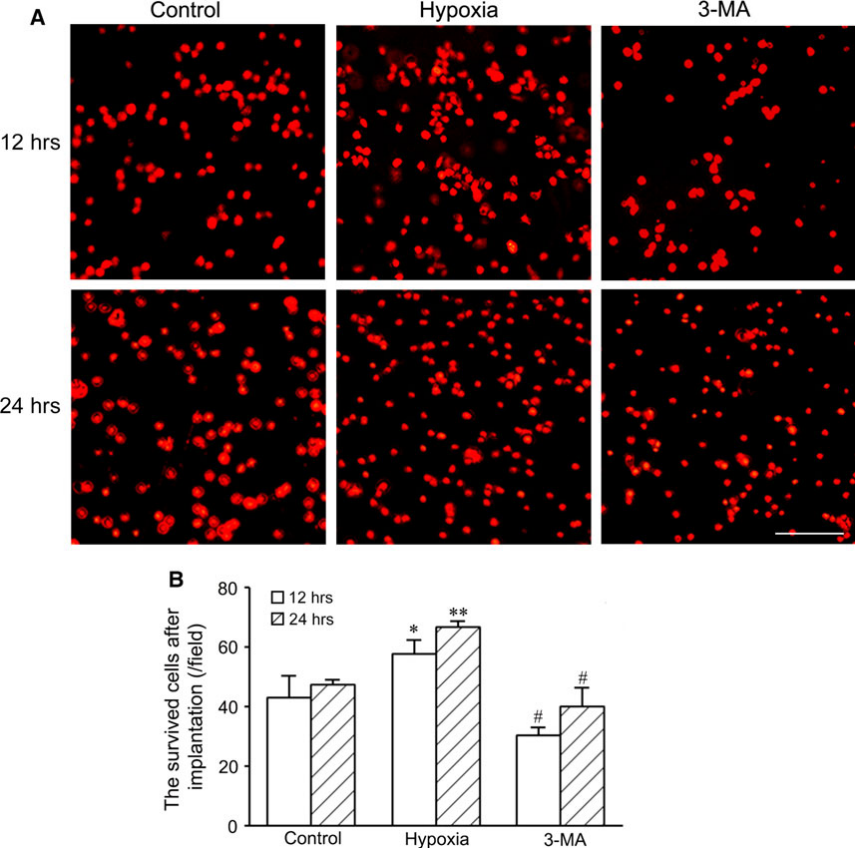
The survived cells in the hypoxia‐pretreated cells after implantation into abdominal pouches. (**A**) Dil‐labelled cells at 12 and 24 hrs after implantation. The cells seed on the polyethylene terephthalate membrane were treated with hypoxia before implantation into the ischaemic pouch of the abdominal wall. The survived cells in hypoxia‐preconditioned cells are more than in control group. In 3‐MA group, the survived cells decrease. Bar = 100 μm. (**B**) Statistic result of the survived cells after implantation (*n* = 3 per group). *P < 0.05 and **P < 0.01 *versus* control group, #*P* < 0.05 *versus* hypoxia group.

At 12 or 24 hrs after implantation of cell‐loaded membrane into abdominal ischaemic pouch, the survival of Dil‐labelled cells was examined. The survived cells in the cells preconditioned with hypoxia (1% O_2_ for 2 hrs) were more than those in control group. After autophagy was inhibited with 3‐MA, the survived cells in hypoxia‐preconditioned cells decreased significantly (Fig. 5).

### Restoration of blood perfusion after transplantation of hypoxia‐preconditioned cells

**Figure 6:**
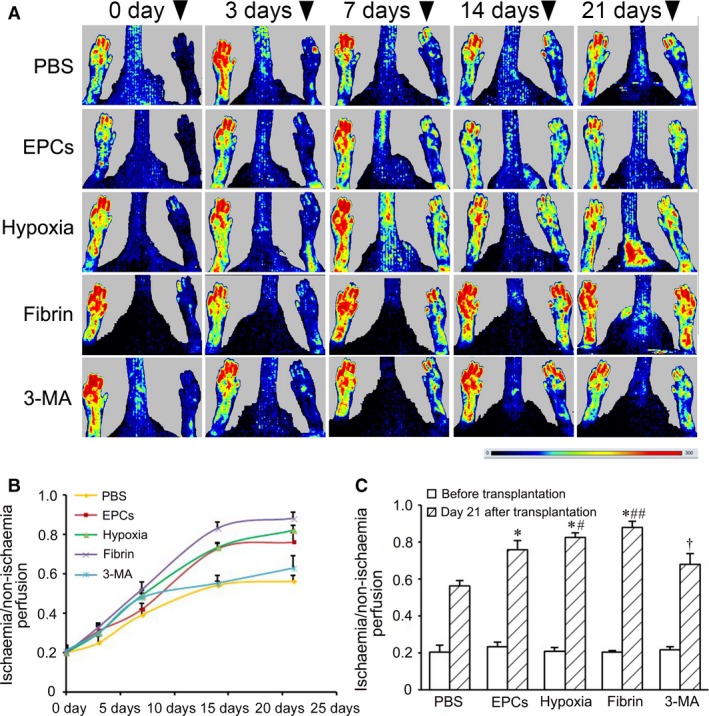
Improvement of blood perfusion in the ischaemic hindlimbs after cell transplantation. (**A**) Typical images of laser Doppler analysis of blood perfusion. At day 0, 3, 7, 14, 21 after ligation of the femoral artery, blood perfusion in the paws of the hindlimbs was examined with a laser Doppler perfusion image analyser. Arrowheads point to the ischaemic limbs. Bar: dark blue represents low‐to‐no flow, red represents high blood flow. (**B** and **C**) Statistic results of blood perfusion at different time‐points (**B**) and day 21 (**C**) after cell transplantation (*n* = 4 per group). *P < 0.01 *versus* control group, #*P* < 0.05 and ##*P* < 0.01 *versus* EPC group, †*P* < 0.05 *versus* hypoxia group. EPC: endothelial progenitor cells.

After ligation of the femoral artery, blood perfusion in the left hindlimb was to <25% of the right hindlimb. In control group, blood perfusion in the paws of the ischaemic hindlimbs restored gradually in the following 3 weeks after ligation of the femoral artery. Compared with control group, restoration of blood perfusion in EPC, hypoxia and fibrin groups was improved. Restoration of blood perfusion in hypoxia group was greater than in EPC group. Transplantation of hypoxia‐preconditioned cells loaded with fibrin improved greatly blood perfusion than transplantation of EPCs only or hypoxia‐preconditioned cells only. Improvement of blood perfusion in 3‐MA group decreased significantly compared with hypoxia group or fibrin group (Fig. 6).

### Enhancement of arteriogenesis and angiogenesis after EPC transplantation

**Figure 7:**
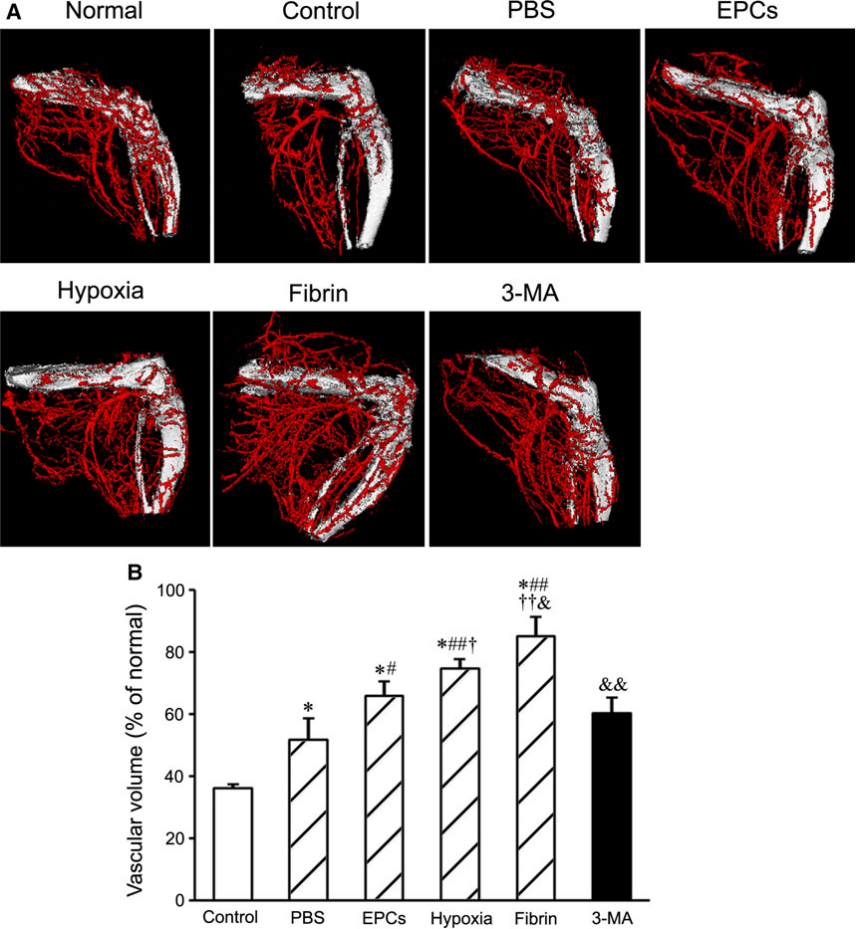
Angiographic analysis of arteriogenesis in the ischaemic limbs after cell transplantation. (**A**) The graphs represent the typical images of the vessels in the ischaemic limbs at day 21 after transplantation. (**B**) Statistic result of the volume of the vessels (*n* = 4 per group). The volume of the vessels is greater in fibrin group than in other groups.*P < 0.01 *versus* control group, #*P* < 0.05 and ## *P* < 0.01 *versus* PBS group, †*P* < 0.05 and ††*P* < 0.01 *versus* EPC group, &*P* < 0.05 and &&*P* < 0.01 *versus* hypoxia group. EPC: endothelial progenitor cells.

The distribution of the vessels in the ischaemic limbs at day 21 after transplantation was assessed with angiography. The collateral vessels in hypoxia group were more than in EPC group. Compared with hypoxia group, density of collateral vessels in fibrin group was higher. Formation of the collateral vessels was reduced in 3‐MA group compared with fibrin group (Fig. 7).

**Figure 8:**
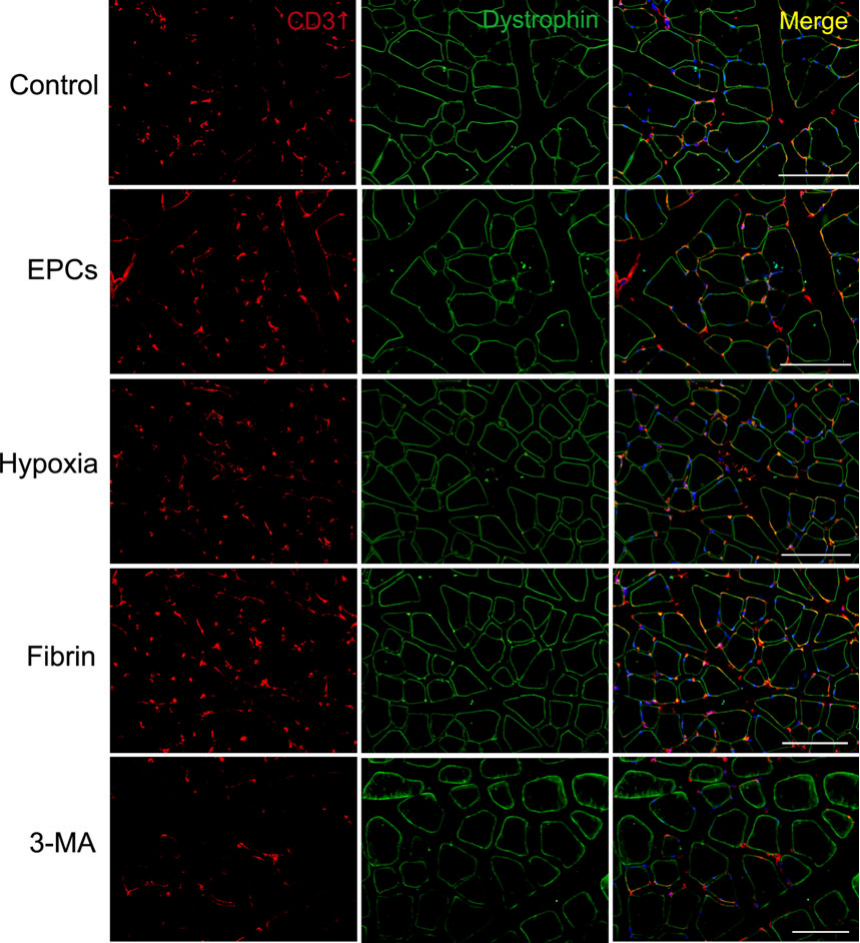
The microvessels in the ischaemic limbs. The microvessels and muscle fibres were demonstrated with CD31 and dystrophin immunostaining, respectively, at day 21 after transplantation. There are more microvessels around the fibres in fibrin group than in other groups. Bar = 100 μm.

The number of the microvessels around muscle fibres in hypoxia group was greater than in EPC group. The density of the microvessels in fibrin group increased significantly compared with hypoxia group. When autophagy of the cells was inhibited with 3‐MA before transplantation, the density of the microvessels decreased after transplantation of hypoxia‐preconditioned cells loading with fibrin (Fig. 8 and Fig. S2). In addition, some GFP‐labelled EPCs expressed CD31 at day 14 after transplantation. GFP^+^CD31^+^ cells were located at the walls of the microvessels (Fig. S3), which revealed direct involvement of EPCs in angiogenesis.

Muscle fibres in the ischaemic limbs were serious injured. The fibre intervals were not distinct. Some of nuclei migrated into the centre from the periphery of the fibres. Although centrally nucleated fibres were detected at day 21 in PBS group, the muscle tissue showed the disorganized pattern. In EPC, hypoxia and fibrin groups, the injured fibres were repaired well.

## Discussion

In this study, we demonstrate that autophagy activated with mild and shout‐term hypoxic preconditioning promotes the survival of the engrafted EPCs in the ischaemic tissue. After treatment with hypoxia, expression of LC3 protein and autophagic structures of the cells increase. The apoptotic cells in hypoxia‐treated cells increase after autophagy is inhibited. In the ischaemic pouch of the abdominal wall and ischaemic hindlimb, viability and survival of hypoxia‐preconditioned cells enhance *via* activation of autophagy compared with the normoxic cells. Recently, we suggest that pre‐adaption of MSCs with hypoxia and ischaemia on the epicardium appears to improve survival and differentiation of the cells after their migration into the hostile environment of the infracted myocardium 34. Hypoxia inducible factor‐1 (HIF‐1) is a key regulator of a broad range of cellular responses to hypoxia such as autophagy and apoptosis 35. It also plays a critical role in cellular bioenergetic adaptation to prevent redox stress and to preserve macromolecular synthesis under hypoxic condition 36. The cells can eliminate the damaged mitochondria *via* autophagy, thereby reducing production of reactive oxygen species and DNA damage and inhibiting the release of pro‐apopototic factors such as cytochrome c 37, 38, 39. Thus, adequate induction of autophagy is beneficial for increasing tolerance of the engrafted cells to ischaemic microenvironment to avoid apoptosis and necrosis.

Our experimental data show that treatment with 1% O_2_ for 2 hrs is an optimal preconditioning for EPC transplantation. Hypoxic preconditions used by other groups are 1–3% O_2_ 15, 1% O_2_ 16 and anoxic 17, 2% O_2_ 18 for 24 hrs to a few days in MSC and EPC transplantation, respectively. Oxygen tension of stem cell niche in the bone marrow is ≈1–7% O_2_ 40. This hypoxic microenvironment is necessary to maintain self‐renew and stemness of stem cells. Application of oxygen concentration above is based on O_2_ levels of the bone marrow. However, severe and sustained hypoxia may induce apoptosis although hypoxia‐induced autophagy is a protective process for cell survival. In our experiment, the apoptotic cells in EPCs treated with 1% O_2_ increase in a time‐dependent fashion as hypoxic treatment prolongs. However, autophagy might not contribute to hypoxia‐induced apoptosis because autophagy induced in this hypoxic condition is mild. Autophagy and apoptosis are main characteristics of cell survival. Therefore, it is reasonable to determine an optimal hypoxia precondition basing on balance between autophagy and apoptosis induced with hypoxia.

Results of our experiments demonstrate that hypoxia‐preconditioned cells enhance collateral growth and angiogenesis significantly and restore microcirculation of the limb and integrity of the muscles effectively after intramuscular transplantation. Angiogenic effects of the transplanted EPCs include differentiation into endothelial cells and production of pro‐angiogenic growth factors and other cytokines which stimulate the existing endothelial cells to form new vessels through paracrine effects 41. EPCs secrete pro‐angiopoietic and pro‐survival factors, including VEGF, basic fibroblast growth factor, insulin growth factor‐1, stromal cell‐derived factor‐1 and interleukin‐8 42. The conditioned medium (CM) derived from both normoxic and hypoxic EPCs provided protective effects on apoptosis of the endothelial cells in hypoxic conditions 43. EPC‐derived CM chemoattracts endothelial cells and displays pro‐angiopoietic activity 42. Results of our experimental reveal that hypoxia stimulates VEGF release and VEGF‐2 expression of EPCs. It is consistent with the results of Akita's group 17. In our experiment, the engrafted cells incorporate into the endothelium of the microvessels around myofibres. Incorporation of EPCs into the vasculature promotes neovascularization in the ischaemic hindlimb 44. It is known that the number of very small embryonic‐like stem cells (VSELs) increases in peripheral blood of patients with CLI. These cells have a potential to participate in revascularization of ischaemic limb 45. Recruitment of VSELs or other stem cells such as MSCs after transplantation of the hypoxia‐preconditioned EPCs remains to be studied.

We have evaluated effectiveness of delivering EPCs with fibrin in the ischaemic limb for the first time. Fibrin gel protects the cells against apoptosis in hypoxia condition. The cells spread well along nanofibres of fibrin. Delivering hypoxia‐preconditioned cells with fibrin gel enhances formation of collateral vessels and angiogenesis. Fibrin is a product following the conversion of fibrinogen in the presence of thrombin. It is structurally similar to extracellular matrix, displaying biocompatible and biodegradable properties. Application of fibrin gel increases retention of the implanted cells in the local musculature 46. Intramuscular injection of fibrin alone may augment neovascularization of hindlimb ischaemia 47, 48. Compared with fibronectin, fibrin enhances viability and cytokine release of EPCs 49. After transplantation into the infarcted myocardium, fibrin promotes survival, migration and cardiomyogenic differentiation of marrow‐derived cardiac stem cells and induces local angiogenesis 27. Constituent and architecture of the extracellular matrix in the ischaemic musculature is changed. Fibrin is effective to improve the local microenvironment. In addition to cytoprotection, fibrin may promote retention, adhesion, proliferation and differentiation of the engrafted EPCs. Stiffness of fibrin gel suitable for application in the musculature of the ischaemic limb will need further investigation.

In summary, this study provides a feasible approach to EPC‐based therapeutic neovascularization in limb ischaemia. We suggest that the contribution of autophagy to hypoxic preconditioning may be beneficial for adaptation of the transplanted EPCs to the ischaemic microenvironment. Determination of an optimal hypoxic preconditioning should be based on balance of autophagy and apoptosis of the hypoxic cells. In view of hypoxia degree of the ischaemic tissue in patients with CLI, it is necessary to estimate O_2_ concentration for hypoxic preconditioning and then determine duration of preconditioning for EPC transplantation.

## Conflict of interest

The authors confirm that there are no conflict of interests.

## Supporting information


**Fig. S1** Effect of autophagy on apoptosis of the cells after treatment with hypoxia for 2 hrs.
**Fig. S2** Statistic result of the density of the microvessels in the ischemic limbs after cell transplantation (*n* = 3 per group).
**Fig. S3** Location of GFP^+^CD31^+^ cells at the walls of the microvessels.
