# Supplementary material for: Hypoxic preconditioning‐induced autophagy enhances survival of engrafted endothelial progenitor cells in ischaemic limb
Source: J Cell Mol Med. 2017 Apr 4;21(10):2452–64. doi: 10.1111/jcmm.13167 (PMC5618704; doi:10.1111/jcmm.13167)
Supplement: Supplementary file 1 — Fig. S1 Effect of autophagy on apoptosis of the cells after treatment with hypoxia for 2 hrs. Fig. S2 Statistic result of the density of the microvessels in the ischemic limbs after cell transplantation (n = 3 per group). Fig. S3 Location of GFP+CD31+ cells at the walls of the microvessels. [file JCMM-21-2452-s001.pdf]

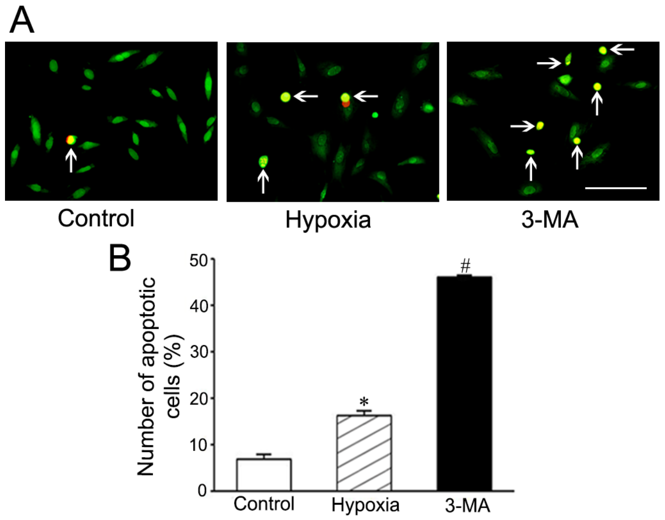

Fig. S1 Effect of autophagy on apoptosis of the cells after treatment with hypoxia for 2 h. (*A*) Images of the apoptotic cells demonstrated with EB/AO staining. After treatment with 3-MA, the apoptotic cells in the hypoxic cells increase. Bar = 100 μm. (*B*) Statistic result of the number of the apoptotic cells in the hypoxic cells. * $p < 0.05$ vs. control group, # $p < 0.05$ vs. hypoxia group.

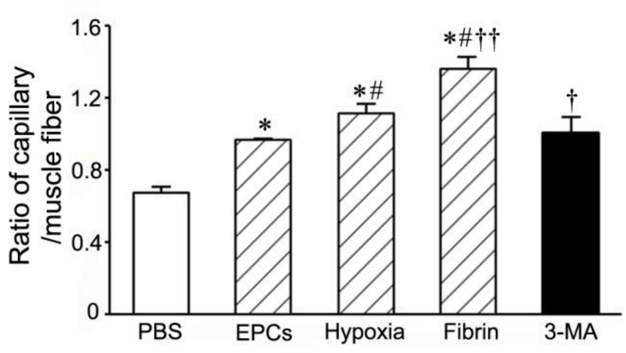

Fig. S2 Statistic result of the density of the microvessels in the ischemic limbs after transplantation. * $p < 0.01$ vs. PBS group, # $p < 0.01$ vs. EPC group, † $p < 0.05$ and †† $p < 0.01$ vs. hypoxia group.

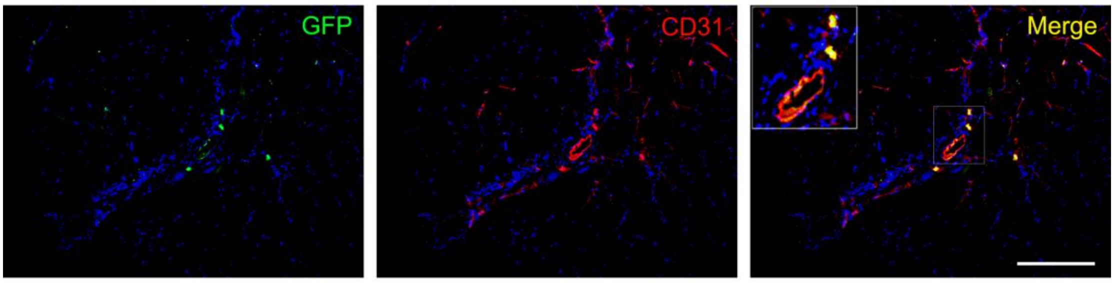

Fig. S3 Location of GFP$^+$CD31$^+$ cells at the walls of the microvessels. At day 21 after transplantation of GFP-labeled EPCs, expression of CD31 on the cells were examined with immunostaining. Some GFP$^+$ cells express CD31 and incorporate into the walls of the microvessels. The large box shows the magnified view of the small box. Bar = 200 μm.
